# Wind Tunnel Performance Tests of the Propellers with Different Pitch for the Electric Propulsion System †

**DOI:** 10.3390/s22010002

**Published:** 2021-12-21

**Authors:** Zbigniew Czyż, Paweł Karpiński, Krzysztof Skiba, Mirosław Wendeker

**Affiliations:** 1 Aeronautics Faculty, Polish Air Force University, 08-521 Dęblin, Poland; 2 Department of Thermodynamics, Fluid Mechanics and Aviation Propulsion Systems, Faculty of Mechanical Engineering, Lublin University of Technology, 20-618 Lublin, Poland; pawel.karpinski@pollub.edu.pl (P.K.); k.skiba@pollub.pl (K.S.); m.wendeker@pollub.pl (M.W.)

**Keywords:** propeller, performance, electric propulsion, propeller pitch, thrust, wind tunnel

## Abstract

The geometry of a propeller is closely related to its aerodynamic performance. One of the geometric parameters of a propeller is pitch. This parameter determines the distance by which the propeller moves forward during one revolution. The challenge is to select a propeller geometry for electric propulsion in order to achieve the best possible performance. This paper presents the experimental results of the aerodynamic performance of the set of propellers with different pitch values. The tests were performed in a closed-circuit subsonic wind tunnel using a six-component force balance. The analyzed propellers were 12-inch diameter twin-blade propellers that were driven by a BLDC (brushless direct current) electric motor. The tests were performed under forced airflow conditions. The thrust and torque produced by the propeller were measured using a strain gauge. The analysis was performed for different values of the advance ratio which is the ratio of freestream fluid speed to propeller tip speed. Additionally, a set of electrical parameters was recorded using the created measurement system. The propeller performance was evaluated by a dimensional analysis. This method enables calculation of dimensionless coefficients which are useful for comparing performance data for propellers.

## 1. Introduction

Unmanned aerial vehicles (UAVs) are now widely used in a variety of applications. Besides the power source, the electric motor, ESC (electronic speed control), and propeller affect the efficient operation of the aircraft. The right choice of a propeller for an aircraft affects flight duration [1,2]. The best solution may be selected based on characteristics provided by propeller retailers or manufacturers, but it is usually difficult or even impossible to do due to a lack of reliable data. Only bench testing the complete propulsion system including the propeller, electric motor, ESC, power supply/battery will give reliable results. Very often, the change of even a single component, e.g., a speed controller significantly influences the performance of a propulsion unit. These differences may concern not only the generated thrust value but also the thermal loads of the propulsion motor, rate of rotational speed increase, rotational speed stability, supply voltage stability, power consumption, efficiency, etc. Various propeller test stand proposals can be found in the literature [3,4,5,6,7]. The propeller theory is well known due to its use for many years as the main propulsion system for fixed-wing aircraft [8]. Theoretical description of small multirotor UAVs is, however, more difficult due to their size, aeroelastic effects, and transverse flow. The performance of a propeller propulsion system is affected by propeller geometry. The key geometrical parameters of a propeller are its diameter, pitch, chord length, local thickness, local camber. A question can be posed about how pitch affects the performance of a propeller driven by an electric motor. The answer to this question is the main objective of this article.

**Figure 1:**
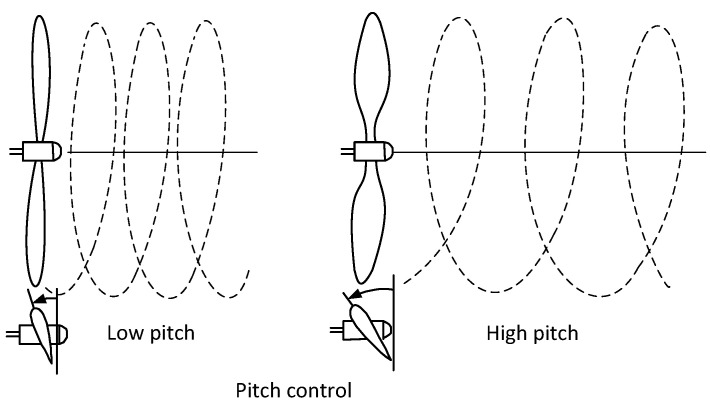
Definition of propeller pitch.

One of the basic geometric parameters is propeller pitch. This parameter is defined as the distance the propeller overcomes in the axial direction during one revolution (Figure 1). Experimental and numerical methods for investigating the performance of propeller blades were presented in [9]. An analysis of the performance data of a small-scale propeller set was carried out by Uhlig (2008) [10] who analyzed the geometric characteristics of the propellers at low Reynolds numbers.

Experimental propeller tests can be performed with forced [11] and without forced airflow [12]. The former allows for performance evaluation during flight (when the relative air velocity is different from zero). These tests are carried out in a wind tunnel using measuring systems that measure forces and pressure distribution. Pressure taps combined with pressure sensors [13] or the PIV (Particle Image Velocimetry) method [14,15] can be used to measure pressure distribution. The issue of testing the small propeller and the rotor in a low-speed aeroacoustic wind tunnel is discussed in [16,17]. A set of propellers at low Reynolds numbers was investigated in [18]. The wind tunnel test method was also used in [19] where dimensionless coefficients of the forces and moments acting on a propeller were analyzed for different wind velocities, propeller angles, and propeller rotational speeds. Performance characteristics and the velocity field of a 16-inch diameter ducted propeller using wind tunnel testing were investigated by Yilmaz (2008) [20]. Chen (2015) [21] analyzed the obtained from wind tunnel testing power and thrust coefficients for a 2-blade counter-rotating propeller. Wind tunnel testing can also be used to validate propeller performance calculation [22,23]. Propeller or airfoil performance under different conditions can be analyzed using simulation methods such as computational fluid dynamics (CFD) [24,25,26] or using computational algorithms using an Euler and Navier-Stokes solver [27]. Numerical simulations can later be experimentally validated [28]. The Particle Image Velocimetry method is useful for an experimental testing of propellers. This method was used in [29] to analyze propeller flow. Based on the wind tunnel tests, the distribution of velocity and its components in the vertical plane passing through the propeller axis were determined for several values of the angle of attack of the tested object for two different values of airflow velocity inside the wind tunnel.

The Blade Element Momentum Theory (BEMT) method can be used to evaluate propeller performance [30,31]. This method is a modification of the Blade Element Theory which also determines the behavior of propellers [32,33]. This method was used by MacNeill (2016) [34] to model low Reynolds number propeller performance. A comparison of modeling using BEMT and CFD calculations is presented in [35]. Mathematical modeling of the aerodynamics of propellers allows for the prediction of its performance and assists in the aircraft design process [36]. It is also possible to combine different analytical methods such as blade-element methods, the momentum theory, and sectional airfoil analysis to evaluate propeller performance [37]. The momentum/blade-element method was used by Gur (2005) [38] to analyze propeller performance at a low advance ratio. A special test rig for testing a wide range of low Reynolds number propellers was presented in [39].

The efficiency of a propeller is determined by the thrust coefficient (1), torque coefficient (2), and propeller power coefficient (3):

(1) $$c_{T}=\frac{T}{\rho \cdot n^{2}\cdot D^{4}}$$

(2) $$c_{Q}=\frac{Q}{\rho \cdot n^{2}\cdot D^{5}}$$

(3) $$c_{P}=\frac{P}{\rho \cdot n^{3}\cdot D^{5}}$$

The dimensionless coefficients for a given air density *ρ* and propeller rotational speed *n* allow the calculation of thrust, torque, and power consumed by the propeller. The propeller diameter *D* in both relationships (Equations (3) and (4)) allows the comparison of propellers of different sizes. A propeller can operate at different flight speeds depending on the type of aircraft. As a result of aerodynamic effects, the propeller generates thrust and torque which must be overcome by engine propulsion. Thrust and torque depend on the airflow around blades, i.e., directly on flight speed. Instead of using coefficients, one can refer to the advance ratio (4), where *v* is the value of undisturbed air velocity. The parameter *J* determines the operating conditions of the propeller. The advance ratio is appropriate for fixed-wing airplanes., Motion for multicopter propellers can be, however, either parallel to the axis of rotation of the propeller or nearly perpendicular to it.

(4) $$J=\frac{v}{nD}$$

Among the theories describing propeller operation, one can distinguish the momentum theory and the blade element theory. A disadvantage of the former is that it does not consider the basic structural parameters of the propeller such as the number of blades, blade shape, type of airfoil, positioning relative to flow. It does not consider the hub either, so it is necessary to know propeller thrust or induced velocity. The latter method involves calculating the aerodynamic forces acting on a blade element of width *dr* located at a distance *r* from the propeller shaft axis, then summing the elementary forces along the blade and multiplying by the number of blades. However, it assumes that the effect of the vortex surfaces generated by blades is neglected. The propeller blade element located at a distance *r* from the axis of rotation and having a width *dr* is affected by an aerodynamic force *dP* which has components *dPx* (5) and *dPz* (6) in the flow-related system.

(5) $$dP_{x}=\rho \cdot c_{x}\cdot v_{0}^{2}\cdot c\cdot dr/2$$

(6) $$dP_{z}=\rho \cdot c_{z}\cdot v_{0}^{2}\cdot c\cdot dr/2$$

where:

ρ—air density [kg/m^3^],
$c_{x}$—drag force coefficient [-],
$c_{z}$—lift force coefficient [-],
$v_{0}$—resultant air velocity [m/s],
dr—blade width [m],
*c*—blade chord [m].

The elementary thrust *dT* (an axial component of *dP*) and the elementary circumferential force *dF* (a circumferential component of *dP*) are of the forms as in (7) and (8), respectively. The angle *γ* used in the equation is the angle of the propeller blade flow defined as the difference between the blade cross-sectional angle and the angle of attack of the blade.

(7) $$dT=dP_{z}\cdot cos\gamma -dP_{x}\cdot sin\gamma$$

(8) $$dF=dP_{x}\cdot cos\gamma -dP_{z}\cdot sin\gamma$$

From the relations (9) and (10), we obtain (11) and (12). *v_T_* refers to the circumferential component of the propeller drag velocity and *v_S_* to the axial component.

(9) $$cos\gamma =v_{T}/v_{0}$$

(10) $$\gamma =v_{S}/v_{0}$$

(11) $$dT=\rho \cdot c_{z}\cdot v_{0}\cdot c\cdot \left(v_{T}-v_{S}\cdot c_{x}/c_{Z}\right)\cdot dr/2$$

(12) $$dF=\rho \cdot c_{z}\cdot v_{0}\cdot c\cdot \left(v_{S}-v_{T}\cdot c_{x}/c_{Z}\right)\cdot dr/2$$

The efficiency of a blade element is described by the relation (13):

(13) η=dT·v/dF·ω·r

Considering the axial and circumferential components, the Equation (14) was obtained:

(14) $$\eta_{el}=\frac{v_{T}-v_{S}\cdot c_{x}/c_{Z}}{v_{S}-v_{T}\cdot c_{x}/c_{Z}}\cdot \frac{v}{\omega \cdot r}=\frac{\left(1-v_{S}\cdot c_{x}/v_{T}\cdot c_{Z}\right)\cdot v_{T}}{\left(1-v_{T}\cdot c_{x}/v_{S}\cdot c_{Z}\right)\cdot v_{S}}\cdot \frac{v}{\omega \cdot r}$$

If the velocity components are defined as (15) and (16) and considering (17) and (18), the result (19) is obtained:

(15) $$v_{S}=v+v_{i}=v\cdot \left(1+a\right)$$

(16) $$v_{T}=\omega \cdot r-v_{v}/2=\omega \cdot r\cdot \left(1-b\right)$$

(17) $$a=v_{i}/v$$

(18) $$b=v_{v}/2\omega r$$

(19) $$\eta =\frac{1-v_{S}\cdot c_{x}/v_{T}\cdot c_{Z}}{1+v_{T}\cdot c_{x}/v_{S}\cdot c_{Z}}\cdot \frac{1-b}{1+a}$$

From the above-mentioned relations, it can be stated that efficiency increases when the lift-to-drag ratio increases and when the coefficient *b* determining the power loss for generating the peripheral velocity components in the propeller stream decreases. Efficiency increases as the coefficient *a* decreases and as the power loss for the induced velocity decreases.

The thrust of a propeller with *i*-blades can be calculated from the expression (20):

(20) $$T=i\int_{0}^{R}dT=\rho \cdot i\cdot \int_{0}^{R}c_{z}\cdot v_{0}\cdot \left(v_{T}-v_{S}\cdot c_{x}/c_{Z}\right)\cdot c\cdot dr/2$$

The torque can be calculated from the expression (21):

(21) $$Q=i\int_{0}^{R}r\cdot dF=\rho \cdot i\cdot \int_{0}^{R}c_{z}\cdot v_{0}\cdot \left(v_{S}-v_{T}\cdot \frac{c_{x}}{c_{Z}}\right)\cdot c\cdot r\cdot dr$$

Considering the above, the propeller efficiency can be determined from the expression (22):

(22) η=T·v/ω·Q

This study is an extended version of the paper entitled “Wind tunnel investigation of the propellers for the unmanned aerial vehicle” presented during the 2021 IEEE International Workshop on Metrology for Aerospace in Naples, Italy [40]. This study was to analyze the performance of a set of 12-inch diameter twin-blade propellers with different pitch values. The propellers were used in combination with a low-power electric motor. Another purpose of the study was to evaluate the performance of the propeller-motor pair for the electric propulsion system. The selected performance coefficients like thrust, torque, power, efficiency, and thrust-to-power ratio were evaluated. The values necessary to calculate the coefficients were obtained from the wind tunnel tests. These tests were followed by the tests without the influence of thrust on the propeller performance. The results were presented at the same conference in the extended version of the paper entitled “Experimental study of propellers for the electric propulsion system” [41].

## 2. Methodology

**Figure 2:**
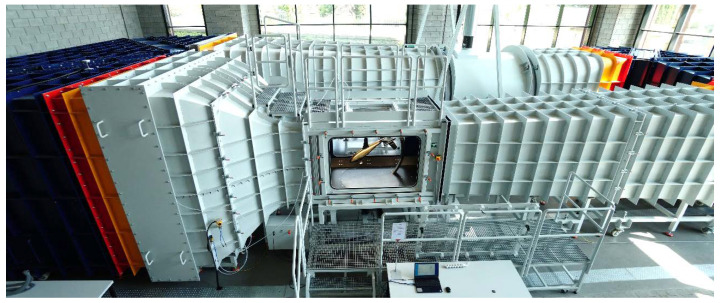
General view of the wind tunnel.

**Figure 3:**
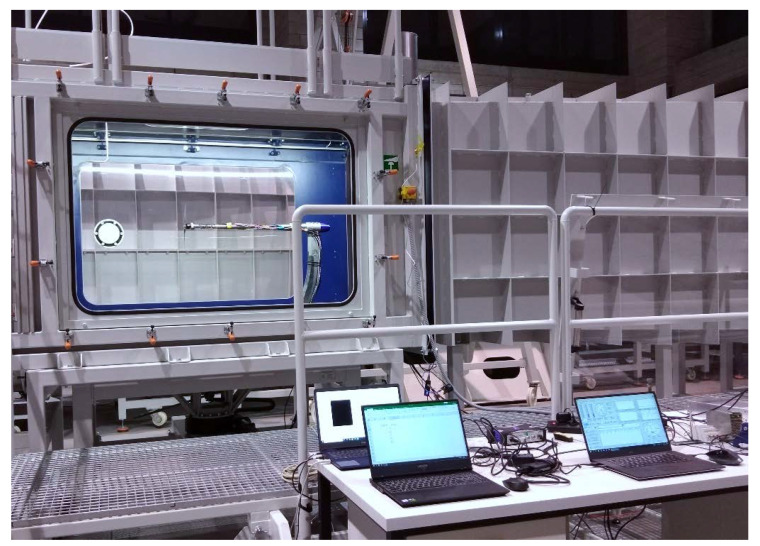
General view of the test stand for propellers.

The research on the selected propellers was conducted on a test stand located in the laboratory of experimental aerodynamics which is a part of the Centre for Innovation and Advanced Technologies of the Lublin University of Technology. The test stand consisted of a six-component force balance placed on a mast located in the central part of the test section. The mentioned components were located inside a 1275 × 1415 mm test section in a closed-circuit subsonic wind tunnel. An axial fan enabled a maximum air velocity of 60 m/s. The maximum turbulence level in the test section did not exceed the value of 0.3%. The angle of deflection of the air stream from the tunnel axis in both planes was less than 0.3°. The view of the wind tunnel is shown in Figure 2, while the view of the test stand and research object in the test section is shown in Figure 3.

**Figure 4:**
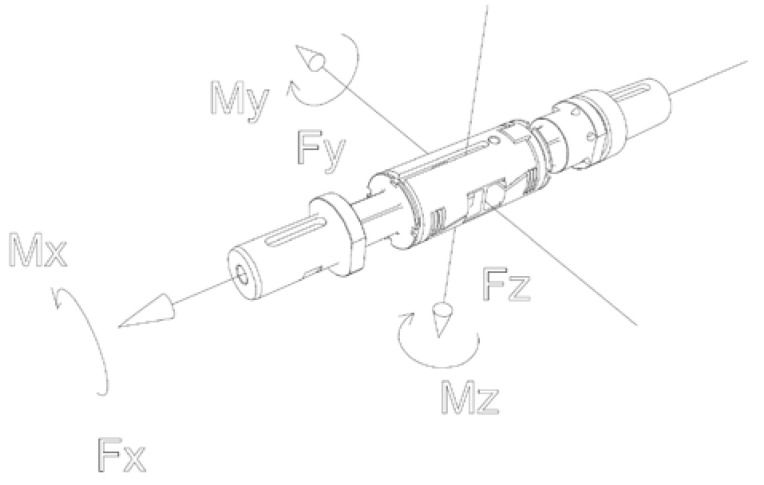
Coordinate system of the FMT625-1b force balance.

Force and torque were measured using a six-component force balance (Figure 4). The average measurement error for the measurement range of the drag force component was 0.11%, and for the moment component *M_x_* was 0.04%. A Prandtl tube with a measurement range of 3–100 m/s was used to measure the airflow velocity. The analog value was converted into a digital one using an Aplisens APR-2000G transmitter. An MX840B HBM measurement amplifier was used to acquire the measurement signals from the monolithic instrumented transducer. For each thrust and torque measurement point, the measurements were made for 5 s at a frequency of 25 Hz, and then the values were averaged. The measured aerodynamic and electrical parameters were averaged from three series of measurements. The data acquisition and parameter control were performed from a computer.

**Figure 5:**
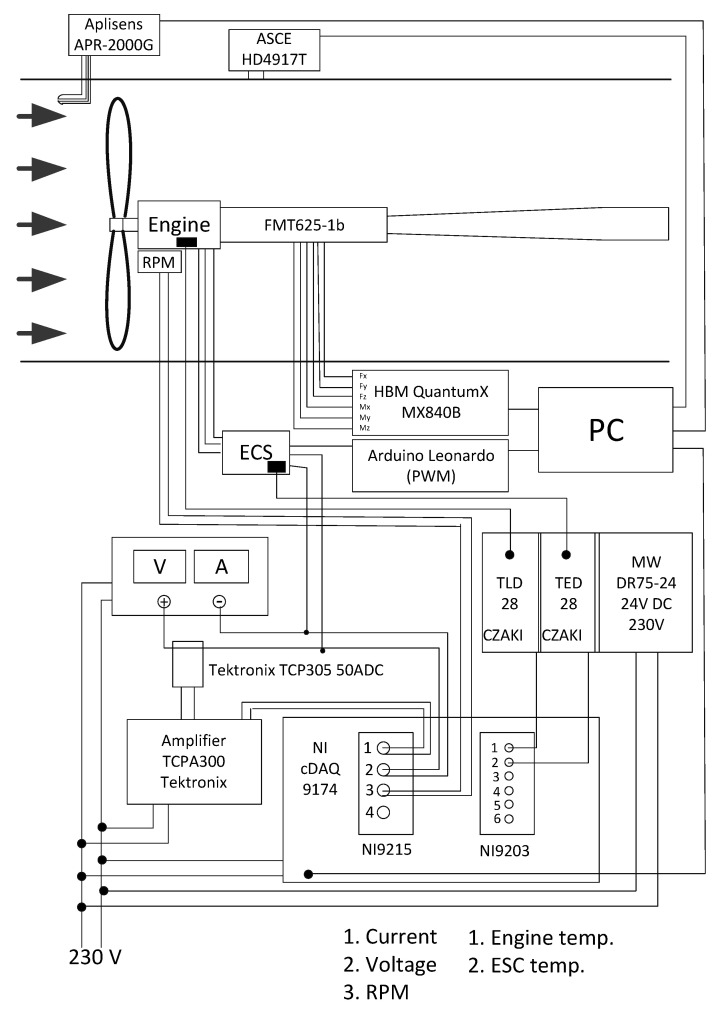
Diagram of the developed control and measurement system.

A special control and measurement system was created to acquire the data from the strain gauge measuring system and measure the electrical quantities. Its schematic diagram is shown in Figure 5. A list of measured parameters and used sensors is given in Table 1. The developed measuring system operated using equipment and software from National Instruments. NI 9215 and 9203 measurement cards installed in a CompactDAQ Chassis cDAQ-9174 module were used. The rotational speed and current were measured using a Bipolar Hall-effect digital position sensor and a Tektronix TCP305 probe with a Tektronix TCP A300 signal amplifier, respectively. At the same time, the supply voltage was measured at one of the ESC (electronic speed control) connectors. To control the temperature conditions, the temperature of the motor winding and ESC was measured using Pt 100 RS Pro sensors. The small size of the sensors required the low thermal inertia of the sensors. An Arduino Leonardo microcontroller was used in the measurement and control line to adjust the PWM (pulse-width modulation) parameter.

Atmospheric conditions were changed during the measurements, so it was necessary to take these parameters into account when calculating air density. The air temperature varied in the range from 25.0 to 29.4 °C, humidity from 36.6 to 38.7%, and pressure from 992.5 to 1018 hPa.

The PWM and the air velocity values were changed during the measurements for the given propeller. Table 2 shows the values of the parameters that were changed during the tests.

**Figure 6:**
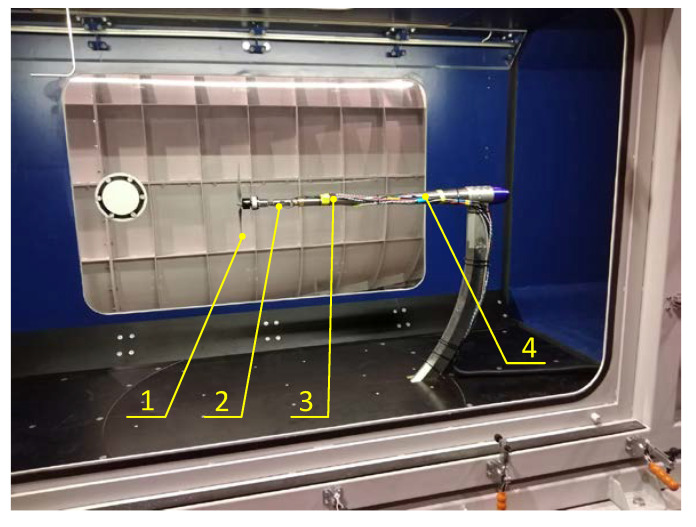
View of the wind tunnel test section with the research object: 1—tested engine-propeller unit, 2—force balance, 3—ESC, 4—sting.

Figure 6 shows the research object inside the test section in the wind tunnel. The tested propeller with a BLDC motor was attached to an adapter. The adapter was then connected to a force balance which was placed at the end of the sting. The sting was attached to a rotating arm that can rotate in two planes. During the experiments, the arm was positioned so that the propeller thrust vector was parallel to the airflow.

## 3. Research Object

**Figure 7:**
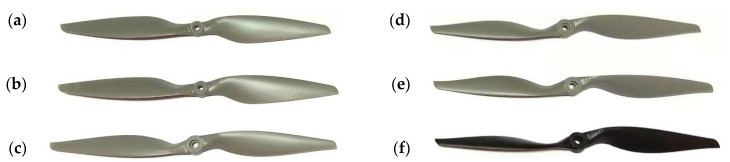
Summary of the tested propellers with different pitches: (**a**) 4.5″, (**b**) 5.5″, (**c**) 6″, (**d**) 8″, (**e**) 10″, and (**f**) 12″.

The test subject was a set of APC propellers (Figure 7). All the propellers tested were 12 inches (0.3048 m) long and had different pitch values. The analyzed propellers had pitch values of: 4.5″ (0.1143 m), 5.5″ (0.1397 m), 6″ (0.1524 m), 8″ (0.2032 m), 10″ (0.2540 m), and 12″ (0.3048 m). Propellers used in this research were made of unreinforced nylon. This material is characterized by the following parameters: tensile strength (75.8 MPa), tensile elongation (above 10%), flexural strength (48.2 MPa), flexural modulus (2.8 MPa). There was a hub in the axis of rotation designed to mount them on the motor shaft. Its thickness was 0.01067 m, and its hole diameter was 0.00635 m.

**Figure 8:**
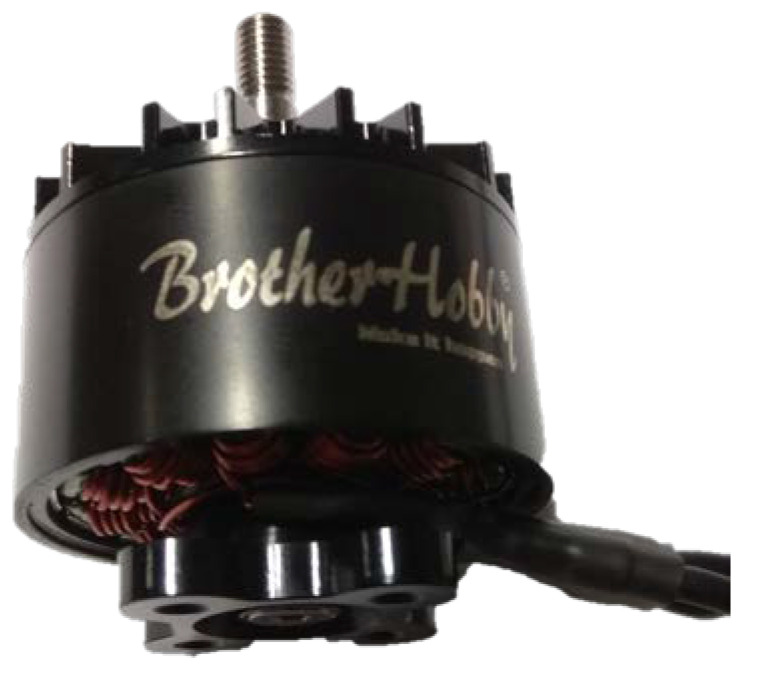
BrotherHobby Tornado T5 3115 electric motor [42].

The propellers were driven using a Tornado T5 3115 electric motor from BrotherHobby (Figure 8). It is a BLDC type unit equipped with an electrically controlled commutator. The motor is designed for the propulsion of light unmanned aerial vehicles. It has a threaded shaft and mounting holes. Its basic technical parameters are shown in Table 3. An ESC ReadytoSky 40 A OPTO and a Mean-Well-RSP-3000-24 power supply were used to power the motor. The nominal supply voltage was set at 22.2 V.

## 4. Results and Analysis

The thrust and torque generated by the given propeller were obtained as a function of the given airflow velocity ranging from 0 to 25 m/s for the defined PWM parameters, i.e., 40%, 60%, 80%, and 90%. The power system parameters like voltage and current were also recorded. Dimensionless coefficients for thrust, torque, power, and efficiency were calculated from the results obtained and in line with the Equations (1)–(3). The thrust-to-power ratio *T_p_* was also calculated.

For each measurement point, these coefficients were expressed as a function of the advance ratio *J* which was calculated from the Equation (4). The power *P* was the product of the measured voltage *U* and current *I* (23):

(23) P=U·I

The propeller efficiency was calculated from the Formula (24). It was calculated as the quotient of the airflow power generated by the propeller and the electrical power supplied to the propulsion unit. The airflow power was calculated as the product of the measured thrust and the velocity of the airflow.

(24) $$\eta =\frac{T\cdot v}{P}$$

**Figure 9:**
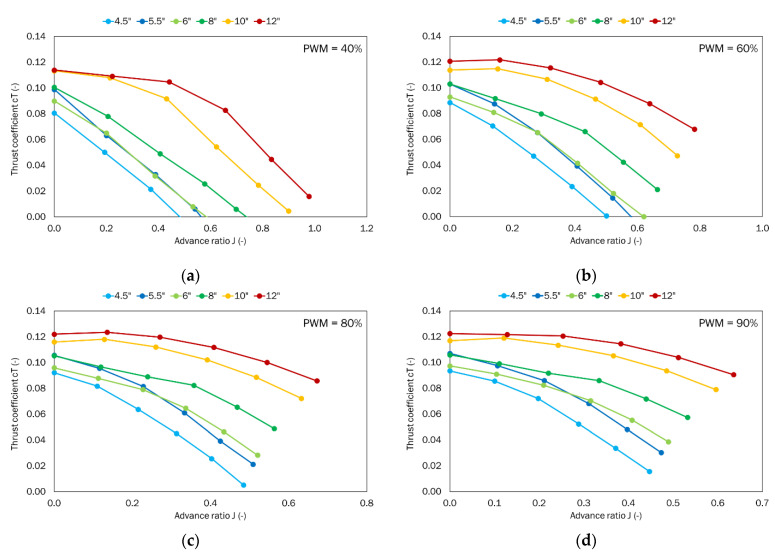
Thrust coefficient as a function of the advance ratio for the tested set of propellers for the defined PWM values: (**a**) 40%; (**b**) 60%; (**c**) 80%; (**d**) 90%.

The first analyzed parameter was the thrust coefficient as a function of the advance ratio (Figure 9). The obtained results are presented for four defined PWM values. As the PWM value increased, the value of propeller rotational speed increased. It was observed that as this parameter increased, the values with a lower advance ratio were obtained. In general, the advance ratio for all the cases considered did not exceed the value equal to 1. For the low rotational speed, i.e., PWM = 40%, the propeller with the smallest pitch had the lowest thrust of all the tested propellers. Increasing the pitch resulted in a gradual increase in thrust. The highest thrust was achieved by the propeller with the largest considered pitch. The increase in pitch simultaneously resulted in curves shifting towards the higher advance ratio and thrust coefficient. The characteristics for the 4.5″, 5.5″, 6″, and 8″ propellers had an approximately linearly decreasing trend. For the propellers with the largest pitch, a slight difference was observed for the small values of the advance ratio, and, in addition, the 5.5″ and 6″ pitch propellers achieved a very similar thrust. The differences occurred for the largest and smallest values of the advance ratio.

**Figure 10:**
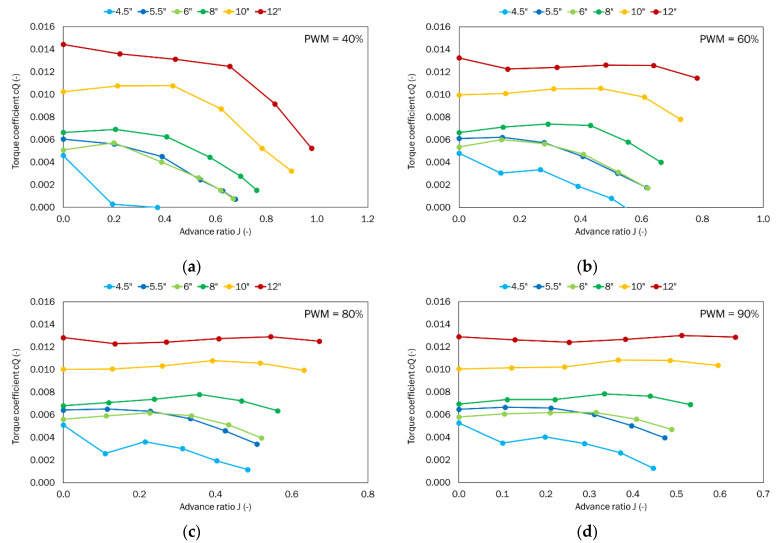
Torque coefficient as a function of the advance ratio for the tested set of propellers for the defined PWM values: (**a**) 40%; (**b**) 60%; (**c**) 80%; (**d**) 90%.

The next analyzed parameter was the torque coefficient (Figure 10). Similar to the thrust coefficient, the highest torque coefficient was obtained for the propeller with the largest pitch (12″). For such a propeller, the torque coefficient decreased with decreasing propeller pitch. For PWM equal to 40%, the highest value of 0.0144 was obtained for the case with no airflow and the advance ratio as 0. With the increase in the airflow velocity, the torque coefficient decreased up to 0.0052 at the advance ratio of 0.98. For PWM equal to 60%, the highest value of 0.0133 was obtained for the case with no airflow where the advance ratio is 0. With the increase in the airflow velocity, the value of the torque coefficient did not decrease in the whole considered range. However, at the advance ratio of 0.78, a value of 0.0115 was obtained. For the PWM of 80%, at the advance ratio of 0, a torque coefficient of 0.0128 was obtained. This is not the highest value, as 0.0129 was obtained at the advance ratio of 0.55. For the maximum PWM of 90%, there was little difference in the torque coefficient as a function of the advance ratio. As in the case of thrust coefficient, propellers with similar pitch, i.e., 5.5″ and 6″ showed comparable values of the torque coefficient. Not all propellers can operate over such a wide airspeed range. The propeller with the lowest pitch produced a positive torque coefficient for an advanced ratio of 0.37 at 40% PWM and 0.6 at higher PWM values. Beyond these extremes of airflow velocity in the wind tunnel, the propellers would generate negative torque, i.e., they would be driven more by the kinetic energy from the flowing air. The most important for the performance are the characteristics obtained for the highest PWM, i.e., 90%. In this case, the propellers with the largest pitch, i.e., 8″, 10″, and 12″ show very small changes in the torque coefficient. However, these propellers apply different loads to electric motors. By comparing the torque coefficient values at the largest advance ratio, it is possible to determine the amount of reduction in the torque coefficient compared to the 12″ propeller. Reducing the propeller pitch to 10″ resulted in a torque reduction of 19%. Further decreasing the pitch to 8″ reduced the torque by almost half (46%). Subsequent propeller changes to the smaller pitch values reduced torque by 64%, 69%, and 90%, respectively.

**Figure 11:**
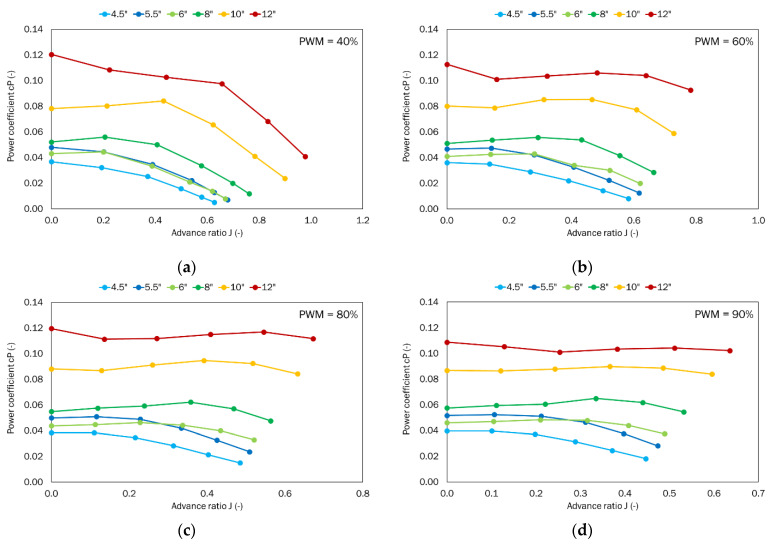
Power coefficient as a function of the advance ratio for the tested set of propellers for the defined PWM values: (**a**) 40%; (**b**) 60%; (**c**) 80%; (**d**) 90%.

Next, the power required to drive the propeller was analyzed (Figure 11). Power strictly depends on torque and rotational speed, so the presented characteristics are similar to the torque coefficient characteristics, but the values obtained were different.

Similar to the previously analyzed parameters, the highest power coefficient was obtained for the propeller with the largest pitch (12″). For such a propeller, the power coefficient decreases as the propeller pitch decreases. For PWM equal to 40%, the highest value of 0.1203 was obtained for the case with no airflow where the advance ratio is 0. As the airflow velocity increases, the power coefficient decreases to 0.0408 at the advance ratio of 0.98. For PWM equal to 60%, the highest value of 0.1127 was obtained for the case with no airflow and the advance ratio as 0. As the airflow speed increases, the power coefficient value does not tend to decrease over the entire considered range, but at the advance ratio of 0.78, a value of 0.0927 was obtained.

For PWM equal to 80% and the advance ratio equal to 0, the power coefficient was found to be 0.1196. The maximum PWM equal to 90% showed a small difference in the power coefficient as a function of the advance ratio. As in the case of the considered thrust coefficient, the propellers with similar pitches, i.e., 5.5″ and 6″ show comparable values of the power coefficient. The most important for performance are the characteristics obtained for the highest PWM, i.e., 90%. In this case, the propellers with the largest pitch, i.e., 8″, 10″, and 12″ show very small changes in the power coefficient. Comparing the power coefficient values at the extreme values of the advance ratio, one can determine the decrease in the power coefficient in relation to the 12″ propeller. Decreasing the propeller pitch to 10″ reduces the power demand by 18%. Reducing the propeller pitch to 8″ results in a reduction of almost half (47%). Subsequent propeller changes to smaller pitches result in power reductions of 63%, 72%, and 82%, respectively.

**Figure 12:**
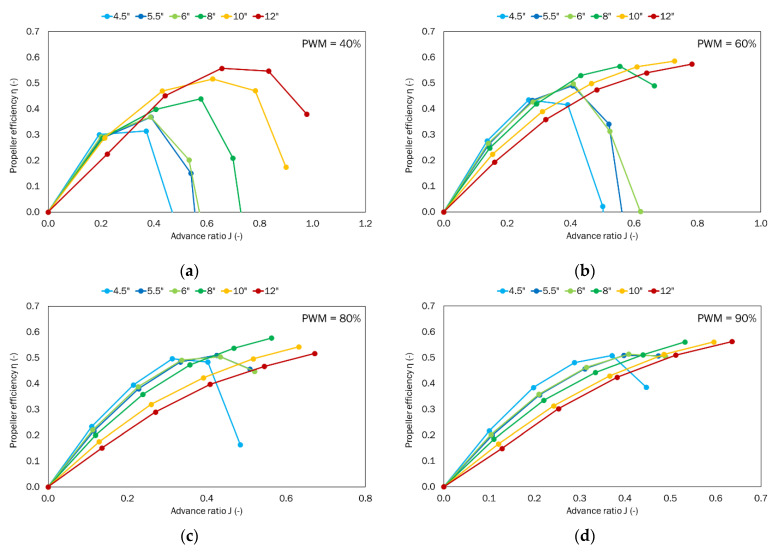
Propeller efficiency as a function of the advance ratio for the tested set of propellers for the defined PWM values: (**a**) 40%; (**b**) 60%; (**c**) 80%; (**d**) 90%.

Another of the analyzed parameters was propeller efficiency (Figure 12). This coefficient defines the most favorable operation range of a given propeller and represents the highest obtained propeller thrust at a given airflow velocity in relation to power consumed. Depending on the PWM set, different characteristics describing propeller efficiency were obtained. For the lowest value of PWM, i.e., 40%, all the obtained characteristics have a distinct extremum. For PWM equal to 60%, two propellers with pitches 10″ and 12″ show increasing efficiency in the whole range as a function of the advance ratio. A similar situation occurs at the next PWM values, i.e., 80% and 90%. For these values, the 8″ propeller shows similar trends. However, in such cases, we cannot determine the best operating point for a given propeller. At these points, we can talk about the maximum efficiency achieved in the considered velocity range. 

For a PWM of 40%, the maximum value of propeller efficiencies varied from 0.31 to 0.56. The point of maximum efficiency moved with propeller pitch toward larger values of the advance ratio. For the propeller with the smallest pitch, the maximum efficiency occurred at an advance ratio of approximately 0.37. This shows that the propeller with the largest pitch, i.e., 12″ has the highest efficiency in the considered conditions (for *J* = 0.66).

In the considered range of airflow velocities for small PWM values, efficiency maxima are observed for all considered propeller pitches. As the PWM value increases, the characteristics for high values of advance ratio (for low propeller speed) do not bend and keep the increasing trend for a higher range of advance ratio.

At PWM 80%, the largest propeller efficiency was achieved by the 8″ pitch propeller. A further comparison was therefore carried out in relation to this value and this propeller. The 12″ propeller in the considered range operated with the maximum value of propeller efficiency lower by 10%. The 10″ propeller in the considered range operated with the maximum value of propeller efficiency lower by 6%, and the other propellers, i.e., 6″, 5.5″ and 4.5″ operated with the maximum value of propeller efficiency lower by 13%, 12%, and 14%, respectively. It can be seen that at such a PWM value the differences are not so significant.

At 90% PWM, the largest propeller efficiency was achieved by the propeller with a pitch of 12″. A further comparison was done in relation to this value and this propeller. The 10″ propeller operated with the maximum value of propeller efficiency lower by 0.2%. The 8″ propeller operated with the maximum value of propeller efficiency lower by 0.3%, and the other propellers, i.e., 6″, 5.5″ and 4.5″ operated at 9%, 9%, and 10%, respectively. It is clear that at this PWM value the differences are even smaller than before and do not exceed 10%. It should be noted that the three propellers with the largest pitch should be tested in this case at higher values of flow velocity to obtain the extrema of the curve describing propeller efficiency. The expected effect would be an increase in the maximum value of this coefficient. This was not necessary in this case due to the fact that the work covered a range of velocities specific to UAV applications.

**Figure 13:**
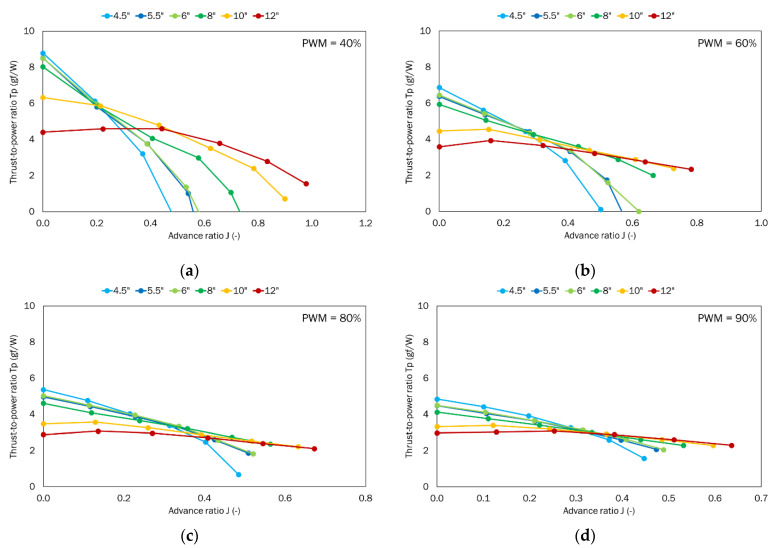
Thrust-to-power ratio as a function of the advance ratio for the tested set of propellers for the defined PWM values: (**a**) 40%; (**b**) 60%; (**c**) 80%; (**d**) 90%.

The last parameter analyzed was the thrust-to-power ratio (Figure 13). This ratio describes the amount of force expressed in gram-force and generated by power expressed in watts. For all propellers except the 12″ pitch one, decreasing characteristics as a function of the advance ratio were obtained. This means that the most gram-force was obtained for the case with no airflow. The exceptions are the 10″ and 12″ propellers.

For propellers from 4.5 to 8″ pitch, the thrust-to-power ratio characteristics are decreasing, i.e., the highest values occur when there is no airflow. For the propellers with pitch 10 and 12″, the characteristics reach their maximum at non-zero values of the advance ratio. An exception is the 10″ propeller, where for PWM = 40% the trend is the same as for propellers with smaller pitches.

For higher airflow velocities, small-pitch propellers generate less force per unit power, while large-pitch propellers operate at a higher thrust-to-power ratio.

## 5. Summary and Conclusions

The conducted research made it possible to calculate the values of dimensionless coefficients of thrust, torque, power, efficiency, and a thrust-to-power ratio. The obtained results allow for the selection of the optimal solution, given the mentioned coefficients as criteria. It was observed that the increase in pitch resulted in the gradual increase in thrust. The highest thrust was achieved by the propeller with the largest considered pitch. Simultaneously, the increase in pitch resulted in the curves shifting towards the higher values of the advance ratio and thrust coefficient. Similarly, to the thrust coefficient, the highest torque coefficient was obtained for the propeller with the largest pitch, i.e., 12″. In this paper, the reduction in the torque coefficient for the tested propellers was specified in relation to the 12″ propeller at the largest advance ratio for the given power demand. At 90% PWM, the largest propeller efficiency (56.2%) was achieved by the 12″ propeller. This is the efficiency of the considered propulsion system and not the efficiency of the propeller itself. The resultant efficiency additionally consists of, e.g., BLDC motor efficiency or speed controller efficiency. It should also be noted that the value of the obtained efficiency does not represent the maximum value for this propeller, because the extremum of the curve describing propeller efficiency was not reached in the studied range. By testing the considered propeller for higher values of the advance ratio, better efficiency is expected. In this paper, it was not necessary to do so, as this range is suitable for typical UAVs. Finally, the thrust-to-power ratio parameter was analyzed. The results show a decrease in the force generated from a unit of power over a range of higher airflow rates, but the large pitch propellers operate with a higher T/Pel ratio over a range of higher airflow velocity rates.

## Figures and Tables

**Table 1:** List of the measured parameters and used sensors.

Parameter	Sensor	Sensor Parameters
Engine speed	Honeywell SS411P bipolar Hall sensor	Sensor type: bipolar Case: TO92 Operate flux: −30…140 Gs Supply voltage: 2.7…7 V DC Temperature range: 40…150 °C
Supply voltage	NI-9215 analog input module with a voltage divider 1:3	Signal levels: ± 10 V, Channels: 4 (differential), Sample rate: 100 kS/s/ch, Simultaneous: Yes, Resolution: 16-bit, Connectivity: Screw-Terminal, Spring-Terminal, BNC
Supply current	Tektronix TCP305 current measurement system with a current probe	AC/DC measurement capabilities, Bandwidth (−3 dB): DC—50 MHz, Risetime: ≤ 7 ns, High-current sensitivity range: 10 A/V, DC (continuous): 50 A, DC Accuracy, Typical (Operating temp. 23 °C ± 5 °C): ±1% of reading
ESC and engine temperature	PT 100 RS PRO SONDE PLATE thermocouple with the programmable measuring Czaki TED 28 transducer	RS PRO PT 100: Type: PT 100—A Class, Probe diameter: 2 mm, Probe length: 10 mm, Maximum temperature: 600°C, Reaction time: 1 m/s Czaki TED 28: Measuring range: 100…800 °C, Processing error: 0.15% or ±0.2 °C, Temperature error/10 °C: 0.05% or ±0.1 °C, Current output signal: 4…20 mA, two-wire, Input-output galvanic isolation, Programmable input signal range, Compensation of the cold ends of the thermocouple
Air temperature, pressure and humidity	Bosch BME280 sensor	Supply voltage: 3.3 V, Interface: I2C and SPI, Temperature range: 40 °C…85 °C, Temperature accuracy: ±1 °C Humidity range: 10% RH to 100% RH, Humidity accuracy: ± 3% relative humidity Pressure range: 300…1100 hPa Pressure measurement error: ±0.25%

**Table 2:** Parameters changed during the tests.

Propeller Pitch (Inches)	PWM (%)	Airflow Velocity (m/s)
4.5	40	0
5.5	60	5
6	80	10
8	90	15
10		20
12		25

**Table 3:** Technical parameters of the tested motor.

Parameter	Sensor Parameters
Weight	110 g with 25 cm SR wires
Motor dimensions (length × diameter)	49.5 mm × 37.5 mm
Rotor	N52H arc magnets
Stator	0.2 mm Kawasaki silicon steel
Shaft dimensions (length × diameter)	16 mm × M5
Maximum power	1150 W
KV factor	640 rpm/V
Maximum voltage	22.2 V

## Data Availability

Data sharing not applicable.
